# A Review of Studies on HIV Pre-Exposure Prophylaxis in Community Pharmacies in States with Restrictive Pharmacist Prescription Authority in the United States

**DOI:** 10.3390/pharmacy12050144

**Published:** 2024-09-24

**Authors:** Hongmei Wang, Dominique Guinn, Xavier Roshitha Ramisetty, Thomas P. Giordano, Ivy O. Poon

**Affiliations:** 1 College of Pharmacy and Health Sciences, Texas Southern University, Houston, TX 77004, USA; hongmei.wang@tsu.edu (H.W.);; 2 Department of Pharmacy, Houston Methodist Hospital, Houston, TX 77030, USA; 3 Department of Health Kinesiology and Sports Studies, Texas Southern University, Houston, TX 77004, USA; 4 Section of Infectious Diseases, Department of Medicine, Baylor College of Medicine, Houston, TX 77030, USA

**Keywords:** HIV, preexposure prophylaxis medications, community pharmacy, pharmacy service

## Abstract

Community pharmacies have unparalleled potential to increase access to pre-exposure prophylaxis medications (PrEP) for HIV prevention; however, only 17 out of 50 states in the United States have statewide authority for pharmacists to provide PrEP at community pharmacies. Few studies have reported on how pharmacists overcome the legislative barrier and provide PrEP services in restrictive pharmacy prescription states. The objective of this article is to identify the existing primary literature describing pharmacist PrEP services in the community in states with restrictive prescription authority. Methods: A systemic literature review was conducted to identify the primary literature that involved community pharmacy service and PrEP conducted in states that do not have expanded pharmacist prescriptive authority between 2000 to 2024. Results: Ten publications were identified, describing nine studies, including four interview and survey studies, three intervention reports, and two ongoing clinical trials. None of these studies have a control group. Most pharmacists provide PrEP services in the community through a collaborative practice agreement with a primary care provider. Conclusions: Future clinical studies with randomized controlled designs are required to test novel strategies in the education and implementation of pharmacy-led PrEP services in a community pharmacy setting to increase PrEP access.

## 1. Introduction

### 1.1. HIV Epidemic and Health Disparities

The HIV epidemic is a critical public health problem affecting 39 million people globally [1]. In the United States (U.S.), 37,601 individuals were diagnosed with HIV infection in 2022, with a rate of 13.3 per 100,000 people aged 13 years and older [2]. California has the highest HIV prevalence (138,531 individuals in 2022) of any state or territory in the United States, followed by New York (124,940), Florida (120,385), Texas (105,365), and Georgia (60,902) [2,3].

Disparities exist in the prevalence and mortality of HIV infection in the U.S.; those aged between 25 to 34 years, self-reported as Black and Hispanic/Latino and male, are associated with the highest rates of HIV diagnosis [2]. In 2022, Black people represented 42% of deaths among people living with HIV aged ≥ 13 years, the highest compared to any other race and ethnicity, which is a 15% increase compared to data from 2018 [3].

The top four transmission categories reported are male-to-male sexual contact (MMSC) (25,193 cases in 2022), heterosexual contact (8380 cases), injection drug use (2621), and both male-to-male sexual contact and injection drug use (1323) [2]. The clinical practice guideline published by the U.S. Public Health Services highlights the need to expand strategies for HIV prevention for the young MMSC population of all races and ethnicities, as well as and Black heterosexuals with a high risk of HIV infection [4].

### 1.2. Preexposure Prophylaxis (PrEP) Medications

Currently, there are three pre-exposure prophylaxis (PrEP) medications approved by the Food and Drug Administration (FDA) in the U.S. [4,5]: oral emtricitabine (F) 200 mg with tenofovir disoproxil (TDF) 300 mg (US brand name: Truvada); oral emtricitabine 200 mg with tenofovir alafenamide (TAF) 25 mg (US brand name: Descovy); and carbotegravir extended-release injection (US brand name: Apretude) [4,5]. F/TDF is FDA-approved for men and women, and F/TAF for men and transgender women in reducing the risk of sexual HIV acquisition [4]. Both F/TDF and F/TAF are administered orally once daily. Oral PrEP is highly effective in reducing the risk of HIV and providing protection up to 99% from sexual intercourse with a positive person and at least 74% among injection drug users. However, its efficacy weans as adherence to the daily administration decreases, with only 76% protection for those who take it twice a week [4]. Cabotegravir is the latest drug approved by the FDA for PrEP to reduce sexually acquired HIV infection [5]. It is an extended-release injection administered once a month for the first two months and every two months thereafter. 

### 1.3. PrEP Uptake and Challenges

Recognizing that PrEP is a key strategy to reduce HIV transmission, the Ending the HIV Epidemic (EHE) initiative sets the goal of having at least 50% of people for whom PrEP is indicated receive it by 2030 [6]. The uptake of PrEP has been low. It is estimated that only 30.1% of those with indications for PrEP received it in 2021 [7]. Despite the fact that HIV is more prevalent in Black people, only 11.1% of those who meet the criteria were prescribed PrEP; this is in contrast to 20.5% of Hispanic/Latino people and 77.9% of White people [7]. Previous research studies have identified barriers and challenges in PrEP prescribing and adherence, including lack of access to a medical office to receive a prescription and follow-up care, inadequate health insurance coverage, concerns about side effects, high levels of perceived anti-HIV and anti-gay stigma, homophobia, and transphobia [8,9].

### 1.4. Pharmacist’s Role Expansion to Increase PrEP Uptake in High-Risk Populations

Pharmacy-based network expansion is one of the most viable solutions to increase PrEP uptake, as recommended in the Toward PrEP Access for All report published by the Southern AIDS Coalition [8]. The uptake of PrEP in the “deep south” of the US, such as Florida, Louisiana, Georgia, and Texas, is further hindered because of more rural and suburban communities with limited access to PrEP and travel time of up to an hour to receive PrEP prescription (PrEP desert). Community pharmacies are undeniably the most accessible option because 88.9% of Americans live within five miles of a pharmacy and many pharmacies have extended hours of operation [8]. It is estimated that 76.5% of pharmacies in rural regions are franchises or independent pharmacies, making independent pharmacies an ideal setting to implement HIV preventive services, especially in rural populations [10]. However, in the U.S., PrEP medications are prescription products. Traditionally, an individual with a PrEP indication would be required to visit a physician to receive a PrEP prescription that can be filled out at a pharmacy, which is a two-step and time-consuming process. To overcome this barrier, legislation has approved an expanded authority for pharmacists to provide PrEP or post-exposure prophylaxis (PEP) to patients at community pharmacies in 17 states and receive reimbursement for their services in some states [11]. Ironically, none of the deep-south states are on the list of 17 states with authority, where the HIV diagnosis rates are high and the access to PrEP is limited. Additionally, few studies have reported strategies to overcome this legislative barrier in implementing PrEP services provided by pharmacists in the community in the states where pharmacist prescriptive authority to PrEP is restricted.

## 2. Methods

**Figure 1:**
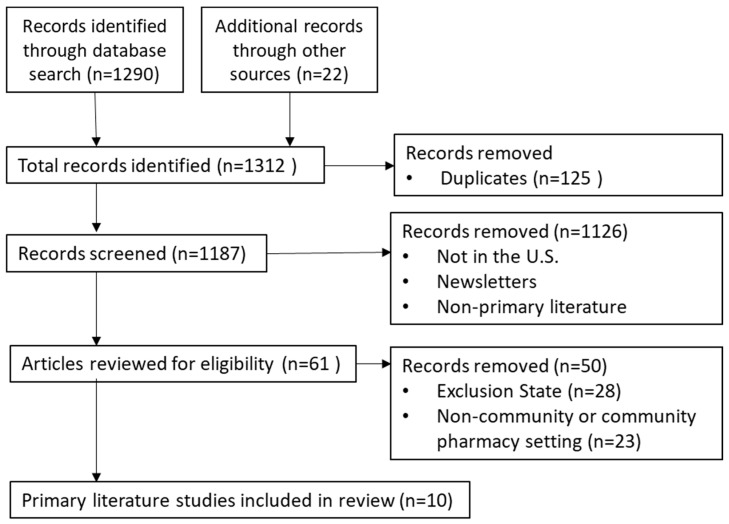
Literature search diagram.

The objective of this literature search was to identify the existing primary literature (original research articles, reports, conference papers) describing pharmacist PrEP services in the community in states with restrictive prescription authority. The MESH search terms “community pharmacy services”, “pharmacies”, “HIV”, and “pre-exposure prophylaxis” were used to search PubMed, Cochrane Library, Medline (Ovid), Scopus, Embase, ClinicalKey, and CINAHL Plus databases. The search terms could be mentioned in the articles’ title, abstract, or body from 2000 to 2024. The search terms were entered into each database using the Boolean operator “AND” in two combinations: “community pharmacy service AND HIV AND pre-exposure prophylaxis”, or “pharmacies AND HIV AND pre-exposure prophylaxis”. Additional searches were conducted through Google Scholar and the clinical trial registry [12]. This literature search was conducted from May to June of 2024. Search results were imported into the reference manager Zotero 6.0 [computer software] [13]. Articles with settings in a pharmacist prescription authority restrictive state to PrEP were included (Alabama, Alaska, Arizona, Connecticut, Delaware, Florida, Georgia, Hawaii, Indiana, Iowa, Kansas, Kentucky, Louisiana, Maryland, Massachusetts, Mississippi, Missouri, New Hampshire, New Jersey, New York, North Carolina, Ohio, Oklahoma, Pennsylvania, Rhode Island, South Carolina, Texas, West Virginia, Wyoming, District of Columbia, American Samoa, Guam, Northern Mariana Islands, Puerto Rico, Virginia Islands). Articles based on the other states where pharmacists have some authority to prescribe PrEP were excluded (California, Colorado, Idaho, Illinois, Maine, Nevada, New Mexico, Oregon, Utah, Virginia, Illinois, Michigan, Minnesota, Montana, Nebraska, New Mexico, North Dakota, South Dakota, Tennessee, Vermont, Washington, and Wisconsin Arkansas). Studies outside the United States and not in the community or community pharmacy setting were excluded. Figure 1 shows a search flowchart describing how articles were chosen for inclusion in this review. There was no restriction on article types screened, but only the primary literature (e.g., original studies, evaluation reports, qualitative reports) was eligible to be reviewed. Each article’s title and abstract were reviewed for relevance using the Zotero group files.

## 3. Results

Our review yielded ten original papers that fit the eligibility criteria of primary literature studies on HIV PrEP and pharmacy services in the community setting in pharmacist prescriptive authority restrictive states [14,15,16,17,18,19,20,21,22,23]. We have grouped the ten studies into three main categories—surveys and interviews [14,15,16,17], intervention reports [18,19,20,21], and clinical trials in progress [22,23]—and provided summaries highlighting each study in detail.

### 3.1. Surveys and Interviews

Table 1 shows the four survey and interview studies conducted to investigate pharmacists’ readiness, knowledge, and acceptance in expanding the practice to provide PrEP service in the community setting. Burns CM et al. surveyed South Carolina pharmacists’ readiness to prescribe PrEP (*n* = 150 pharmacists) among a cohort of pharmacists practicing in various settings, from retail and hospital to academia [14]. Although South Carolina is not a state where the pharmacist has the authority to prescribe PrEP in the community setting, it is one of the main states in the “*deep south*” and is targeted by the U.S. Department of Health and Human Services EHE initiative due to its high rates of HIV in rural areas. It is estimated that more than 10,000 people are eligible for PrEP in South Carolina, but only 11.7% received PrEP. The survey aimed to determine the pharmacists’ readiness, acceptance, and feasibility for expanding the scope of practice to prescribe PrEP should the prescriptive authority become legal in the future. The investigators distributed an online 43-question descriptive questionnaire through an email link from the University of South Carolina Kennedy Pharmacy Innovation Center. A total of 2680 unique emails were sent out and 129 pharmacist participants completed the entire survey. Participants represented a variety of pharmacy practices, including 25% in small towns, 25% urban, 24% suburban, and 11% rural. Among the respondents, 64% reported being comfortable with obtaining a sexual health history from patients, and 49% had experience with sexual health discussions with patients. A total of 67% had provided care for MSM, and 83% felt at least somewhat comfortable providing care for MSM population. Despite most pharmacists reporting being at least slightly comfortable with HIV risk reduction counseling, more than half (67%) did not know how to manage a patient on PrEP, and 60% reported being somewhat ready to provide PrEP services. Potential barriers reported were the cost, lack of time, staffing limitations, the liability of providing a new service, and the lack of comfort in prescribing PrEP. Overall, pharmacist participants believed that PrEP was beneficial, and that the pharmacy was a feasible and acceptable location at which to implement PrEP services. This survey was the first to determine South Carolina pharmacists’ perception of prescribing PrEP, a new and potentially expanded role of pharmacists. The study highlighted the need for educational programs to prepare pharmacists to provide PrEP services. One limitation of the study was that the response rate was low, as expected from an online survey. A strength of the study was that the participants’ demographics resembled that of the pharmacist population nationally.

Crawford ND et al. published a semi-structured, in-depth interview study to investigate the interests of key stakeholders in implementing HIV, STD, and PrEP screening in the community pharmacy setting among a cohort of pharmacists and MSM in Atlanta, Georgia (*n* = 14) [15], a state with restrictive policies for pharmacists prescribing PrEP. The study design involved qualitative analysis of semi-structured interviews of pharmacists and MSM clients. Participants were recruited from pharmacies located in the zip codes with a high prevalence of HIV within the metropolitan Atlanta area. Interviews were conducted with 8 MSM and six pharmacist participants. The median age of MSM participants was 30.5 years old (range 25–54), and 50% were self-reported as Black. The study found that both MSM and pharmacist participants strongly supported the implementation of HIV and PrEP screenings in the community pharmacy setting because the pharmacies were more accessible than the physician’s offices. Pharmacy staff training on HIV and PrEP counseling and private consultation spaces were perceived as two essential areas to focus on in implementing community pharmacy-based PrEP services. One strength of this study was the study population, explicitly targeting the MSM group, which had been the population with the highest rates of HIV diagnosis, and the study location targeted community pharmacies where HIV rates were high. The in-depth interviews using the Consolidated Framework for Implementation Research (CFIR) provided invaluable insights by key stakeholders (pharmacists and MSM clients) to guide implementation strategies in the future. A limitation of the study was the small sample size in urban settings; thus, the results might not have represented those pharmacies in rural settings.

A subsequent qualitative study, conducted by the same investigator group as Crawford ND et al., determined the perception of pharmacists and pharmacy technicians [16]. The investigators utilized a similar study design and semi-structured in-depth interviews to understand the facilitators and barriers to implementing HIV and PrEP screening in an urban community pharmacy setting. A total of six pharmacy technicians and seven pharmacists were interviewed. Overall, pharmacy technicians and pharmacists showed high concordance in supporting the implementation of community-based PrEP services. Pharmacy technicians expressed more concerns about patient privacy, community education regarding PrEP, and the importance of having allotted time to perform PrEP screenings and activities. This study added to the literature by reporting another important stakeholder’s readiness to implement the new PrEP service in community pharmacies. The pharmacy technicians are direct and customer-facing and have crucial roles in the community pharmacy settings in assisting the pharmacists with dispensing prescriptions, solving insurance claims, answering clients’ questions, and managing inventory. Therefore, it is vital to garner their support and needs before implementing the new service.

Shaeer KM conducted a cross-sectional survey to determine the Florida pharmacists’ experience, knowledge, and perception of PrEP [17]. The study, conducted in another restrictive state, successfully received 225 pharmacists’ survey responses. The results showed that 63% were unaware of the Centers for Disease Control and Prevention PrEP guidelines, 71% felt that they did not have sufficient knowledge to counsel patients on PrEP, and 47% reported not comfortable counseling patients with PrEP. The authors concluded that educational programs were needed to address the gaps in knowledge and readiness in pharmacist-led PrEP services. One limitation of this study was that this survey was conducted more than ten years ago. Since then, pharmacists’ knowledge and readiness in PrEP counseling may have evolved as more PrEP medications have been approved and new CDC guidelines have been issued.

### 3.2. Intervention Reports

A summary of the three intervention reports included in this review is provided in Table 2. The Iowa TelePrEP program was first reported by Hoth AB et al., and the program’s expansion was discussed in detail by Chasco EE et al. [18,19]. Hoth AB and Chasco EE et al. described the Iowa TelePrEP program as a statewide PrEP delivery model to expand PrEP access to rural and small urban communities [18,19]. In Iowa, no statute nor statewide standing order gives pharmacists the authority to initiate PrEP independently. However, pharmacists have the legal authority to enter into a collaborative practice agreement (CPA) with a primary care provider to initiate PrEP [24]. The CPA in Iowa does not need to be patient-specific. This exciting program started in 2017 with three public health partners (PHP) and expanded to 12 PHP by 2019. PHP included disease intervention specialist/partner services in regions, local health departments, and non-profit organizations. PHP staff submitted referrals to the TelePrEP program using a standardized screening and risk assessment tool. A patient navigator coordinated the laboratory tests and scheduled an initial telehealth visit with a pharmacist from the University of Iowa. The pharmacists provided a virtual visit, offered counseling and prescribed the PrEP to be delivered by mail or picked up at pharmacies. The initial phase was successful and expanded statewide. A total of 700 clients were referred by PHP partners, of which 206 (80%) received a pharmacist visit, and 167 clients received a PrEP prescription. The majority of clients were White (83%) and male (95%), with a mix of small urban (54%) and rural (22%) residencies. Most clients were MSM with risk factors (94%). Interviews with PHP staff showed that calls, presentations, and site visits to increase PrEP education and awareness were valued. The PHP staff showed high acceptability to the implementation of the TelePrEP programs. One strength of this report was the statewide implementation of telehealth PrEP counseling and initiation in collaboration with local health agencies. A limitation of this study was the intentional sampling of informants who were interviewed, while others who were referred but did not use the service were not recruited for interviews. Also, there was no mention of medication adherence follow-up.

Khosropour CM et al. reported the implementation of a same-day PrEP service model delivered by a clinical pharmacist in a university-affiliated HIV/STD testing center in Jackson, Mississippi, where the prevalence of HIV is high and the network of clinical PrEP providers is small [20]. Missouri, a state in the southern U.S., has no statute nor statewide standing order that gives pharmacists the authority to initiate PrEP independently. However, the law offers pharmacists broad authority to administer injectable PrEP, and pharmacists can enter a CPA with a primary care provider to initiate PrEP [24]. The CPA must be patient-specific in Missouri. In the study, the pharmacist received referrals of potentially eligible patients with indication for PrEP through the HIV/STD testing center. The pharmacist conducted a medical history review, educated the patient on HIV and PrEP, evaluated medication copayment, and scheduled a follow-up clinic appointment within six weeks. The pharmacist provided a 60-day PrEP medication prescription supply through a collaborative practice agreement with a physician and sent it to the patient’s preferred pharmacy. The result showed that 69 individuals received the service over seven months, with 76.8% Black, over 60% aged 29 years or younger, 55.1% MSM, 65.2% without insurance, and 75.4% utilizing the pharmaceutical company patient assistant program in paying for the PrEP. Among those referred to the pharmacist, all received a PrEP prescription, 77% filled the prescription, and 43% attended the follow-up appointment. The main reason for the loss to follow-up was that the patient was no longer interested in taking PrEP. This study was unique because it described a model of pharmacist-prescribed PrEP in a university-affiliated clinic adjacent to the state health department, allowing for direct access and referral of the target population to the pharmacist for the evaluation of PrEP contraindications. This model differed from the pharmacy-based PrEP service, where the pharmacist provided a 60-day PrEP prescription under collaborative practice agreements (CPA) with physicians. Several limitations were noted in this study: the study sample size was small; the authors did not capture the number of individuals reached for screening for PrEP; the lack of a control group; and unknown medication adherence, even if the patient filled the prescription and attended the follow-up appointment. The study was conducted in a pharmacy within the university-affiliated clinic and not in a community pharmacy setting.

Taliaferro T et al. conducted a pharmacist-led education program for undergraduate students at Howard University in Washington, DC, a city with the highest risk of acquiring HIV in the U.S. [21]. The Principal Investigator (P.I.) of this study is a staff pharmacist at Safeway Pharmacy affiliated with the University of Maryland School of Pharmacy. The intervention includes a 30-minute educational program on HIV and PrEP in small groups. A total of 116 students (95.1% Black, 67.7% female) enrolled and participated, and 102 completed a pre- and post-cross-sessional survey to evaluate the perception of knowledge about HIV prevention and PrEP. The study showed that participants’ perceptions of knowledge on HIV prevention, risk factors, and PrEP were higher after the educational program compared to baseline. This study highlighted the significant role of community pharmacists in public health in HIV prevention, risk factor education, and PrEP awareness. It also pioneered the possibility of a group educational class by a pharmacist with expert knowledge in the HIV and PrEP fields in the community setting. One limitation of this study was the lack of a control group and the self-reported subjective nature of the perception of knowledge.

### 3.3. Clinical Trial in Progress

FINISHING HIV is a hybrid 2 implementation-effectiveness randomized controlled trial (RCT) trial in progress (NCT06406049) supported by the National Institute of Health (NIH) [22]. The goal of the FINISHING HIV trial is to implement the EHE model using a local HIV agency for Latino men who have sex with men (LMSM) and a CVS pharmacy chain in Miami, Florida. Florida has no statute nor statewide standing order that gives pharmacists the authority to independently initiate PrEP; however, pharmacists can enter into a CPA with a primary care provider to provide PrEP service [24]. The CPA does not need to be patient-specific. Study participants are recruited from these two collaborating sites. Inclusion criteria are age 18 to 54 years old, cis-gender male, self-report of one or more Centers for Disease Control and Prevention (CDC) clinical guidelines for PrEP eligibility, self-report of HIV-negative status and willing to test for HIV. Those who self-identify as non-Latinx or who display diminished cognitive capacity are excluded. Recruited participants are randomly assigned to the standard of care (a 15-minute in-person education) or a social network intervention that consists of 2 h of counseling for three sessions on sexual health for HIV prevention and information and resources for PrEP usage. Participants are encouraged to share what they learn with others two times within the next 12 months. The primary outcomes are the number of participants who reported using PrEP, dried blood spot (DBS) tests that confirmed PrEP use, and the proof of PrEP prescription. The secondary outcomes are PrEP knowledge measured via a validated PrEP knowledge questionnaire and PrEP adherence supported by DBS. The target sample size is 624 individuals. This study is one of the largest RCT that investigates the implementation of HIV prevention education strategies in a social network and a community chain pharmacy setting.

Crawford N et al. are conducting a single-group clinical trial in progress (NCT04393935) to investigate utilizing community pharmacies to increase PrEP uptake in a 3-month behavioral intervention in Georgia [23]. Georgia has no statute nor statewide standing order that gives pharmacists the authority to initiate PrEP independently; however, pharmacists can enter into a CPA with a primary care provider to provide PrEP service [24]. The CPA must be patient-specific, identifying each patient for whom the pharmacist is authorized to modify drug therapy. The study design is a single-group intervention with a target sample size of 50 individuals. The target study population is adult Black MSM residing in minority neighborhoods with high rates of poverty in Atlanta, Georgia. Study participants are recruited at two community pharmacy sites and receive a kit of self-administered tests for HIV, rectal chlamydia, syphilis, gonorrhea, and creatinine. A pharmacist or pharmacy technician evaluates the test results. The pharmacist provides a 7-day PrEP prescription and counseling and schedules a follow-up appointment with a PrEP-prescribing physician. Adherence to PrEP prescription is assessed at three months. This is an ongoing clinical trial, and the result is pending.

## 4. Discussion

### 4.1. How Does a Pharmacist Provide HIV and PrEP Services in the Community Setting in Prescriptive Authority Restricted States?

Currently, 17 states in the U.S. have a statewide standing order giving pharmacists the authority to initiate PrEP independently [25]. The other states do not have statewide standing orders, but many state legislatures allow pharmacists to establish a CPA with a primary care physician to provide PrEP service. In this review, we found two intervention reports in Iowa and Mississippi, where pharmacists had successfully implemented HIV testing and PrEP initiation in the community setting through a CPA and received patient referrals through local health agencies [18,19,20]. In both reports, the pharmacists were clinical pharmacists and had allotted time to provide the service; however, the training and credentials of the clinical pharmacists were unknown. In one study, the pharmacist scheduled a follow-up appointment to monitor adherence and discuss medication management, similar to the Medication Therapy Management model [20,26]. One limitation of this review article is that we excluded states with expanded pharmacist prescriptive authorities and thus the article may not represent all pharmacists’ perspectives in the US. States with expanded pharmacist prescriptive authorities do not experience the legislative barrier and may present different perspectives on this matter.

### 4.2. How Do Important Stakeholders Feel about Pharmacists Providing HIV and PrEP Services in the Community Setting in Prescriptive Authority Restricted States?

Overall, qualitative studies reported that the key stakeholders (pharmacists, pharmacy technicians, and patients) were “ready”, “willing”, and “supportive” with respect to implementing PrEP services in community pharmacy settings in South Carolina, Georgia, and Florida [14,15,16]. A common perceived challenge in implementing PrEP in community pharmacy settings includes the lack of knowledge about HIV and PrEP [17], the lack of a private area for counseling in the pharmacy [16], and the lack of time to provide the service [16].

### 4.3. What Gaps Are Yet to Be Filled to Implement HIV and PrEP Services in the Community Pharmacy Setting in Prescriptive Authority Restricted States?

One of the major gaps is that individuals residing in the “PrEP desert” may need to commute an hour to the city center to seek an in-person PrEP provider. PrEP services are provided by multiple healthcare facilities in major metropolitan areas in the U.S., commonly available in community clinics, local health departments, and adult rehabilitation centers, according to hiv.gov [27]. Many of these healthcare facilities serve people experiencing homelessness, substance abuse, and the LGBTQIA+ population of the city; there are fewer in suburban and none in many rural areas [28]. Although PrEP providers are also available through telehealth platforms, the continuity of care in initiation, counseling, and monitoring of PrEP are unknown, and technical barriers and those pertaining to access to smartphones exist. Independent community pharmacies are a feasible location at which to increase PrEP access because about 88.9% of Americans live within five miles of a pharmacy, many pharmacies have extended hours of operation [8], and community pharmacists have built and sustained trust within each community [29]. Independent community pharmacies differ from chain pharmacies in that (1) the pharmacist-in-charge is typically the owner of the store; (2) there are more independent pharmacies in rural communities; and (3) many independent pharmacies have a list of engaged customers to sustain their business [10].

The overall consensus on the need for an educational program for pharmacists is not surprising because HIV PrEP is listed as a Tier 2 topic in the American College of Clinical Pharmacy Pharmacotherapy Didactic Curriculum Toolkit, meaning this is a topic that “should be covered by most colleges”, though not in an extensive manner, to train pharmacy graduates to be proficient in providing direct patient care as in the Tier 1 topics [30]. The first HIV PrEP medication was approved 12 years ago; therefore, community pharmacists who graduated before that time would need continued education on PrEP [4]. Most community pharmacists do not have post-graduate Year 1 or 2 residency training. They will benefit from a Pharmacy-Based HIV Prevention Services certificate training program on sexual health, PrEP, and PEP counseling through the American Pharmacists Association (APhA) [31]. This program provides 12 contact hours of Continuing Pharmacy Education (CPE). Other studies reported the importance of educational and training programs to pharmacists with respect to HIV PrEP and PEP [32,33].

## 5. Conclusions

Our literature review found few studies on the implementation of HIV PrEP initiation and follow-up services by community pharmacists in states where there was no statewide standing order for pharmacists to initiate PrEP. Every example in the current literature is a qualitative study or an intervention report, and none of the studies has a control group. Future studies are needed to explore the utilization of community pharmacists to increase access to HIV PrEP services in states where a collaborative practice agreement is allowed and to investigate the strategies and interventions that are effective in reaching the at-risk population in HIV.

## Figures and Tables

**Table 1:** Interviews and surveys to determine the readiness of community pharmacists in PrEP counseling and role expansion.

Author (Year)	State/Setting	Methods	Results	Limitations
Burns CM et al. (2023) [14]	South Carolina Retail, hospital, independent, community, specialty, academia	43-question online survey through a pharmacist listserv	More than half of pharmacists (*n* = 129) responded ready and willing to prescribe PrEP	Low response rate; only pharmacists in the university’s listserv were invited
Crawford ND et al. (2020) [15]	Georgia Community pharmacies in zip codes with high HIV incidence	Semi-structured interviews of pharmacists and MSM clients	Participants (*n* = 14) reported in supportive of HIV, STI, and PrEP screenings	Small sample size; limited to urban setting
Hopkins R et al. (2021) [16]	Georgia Community pharmacies in zip codes with high HIV incidence	Semi-structured interviews of pharmacists and pharmacy technicians	Community pharmacists (*n* = 7) and pharmacy technicians (*n* = 6) were supportive of implementation of HIV and PrEP screening	Small sample size; perception may differ among other stakeholders
Shaeer KM et al. (2014) [17]	Florida Community pharmacies	In-person and online survey	Pharmacists (*n* = 225) reported limited understanding of PrEP and more education was needed	Perception may have changed after the study period 10 years ago

**Table 2:** Pharmacist intervention reports.

Author (Year)	State/Setting	Methods	Results	Limitations
Holt AB (2019) [18] Chasco EE et al. (2021) [19]	Iowa Statewide public health departments and University of Iowa collaboration	The pharmacists provided counseling and prescribed PrEP through a collaborative practice model	12 public health partners referred 708 individuals over 18 month period; 258 individuals received TelePrEP service; and 167 individuals initiated PrEP	Intentional sampling may lead to bias; did not measure adherence to PrEP
Khosropour CM et al. (2020) [20]	Mississippi An academic affiliated HIV/STD testing center located next to the state health department STD clinic	The clinical pharmacist received patient referrals from a STD clinic, collected medical history, prescribed PrEP through collaborative practice agreements, and provided a follow up appointment	69 patients were referred to clinical pharmacy service over a 7-month period; 80% received a PrEP prescription on the same day; 77% filled the PrEP prescription; and 43% of those who filled the prescription attended a follow up appointment	Small sample size; lack of control group; not in community pharmacy setting
Taliaferro T et al. (2021) [21]	Washington, DC College campus	The pharmacist provided a 30-minute education to college students about HIV prevention and PrEP	Participants (*n* = 102) reported an increase in perception of HIV and PrEP knowledge after the education compared to baseline	Lack of control group; self-reported subjective measure of knowledge

## Data Availability

All data are contained within the article.

## References

[1] UNAIDS 2023 Report. UNAIDS—Global Report 2023. https://thepath.unaids.org/.

[2] U.S. Centers for Disease Control and Prevention AtlasPlus—Map. Explore CDC’s Atlas Plus HIV+Hepatitis+STD+TB+Social Determinants of Health. https://gis.cdc.gov/grasp/nchhstpatlas/maps.html.

[3] (2022). HIV Surveillance Report: Diagnoses, Deaths, and Prevalence of HIV in the United States and 6 Territories and Freely Associated States. https://stacks.cdc.gov/view/cdc/156509.

[4] Centers for Disease Control and Prevention, US Public Health Service (2021). Preexposure Prophylaxis for the Prevention of HIV Infection in the United States—2021 Update: A Clinical Practice Guideline. CDC. https://www.cdc.gov/hiv/pdf/risk/prep/cdc-hiv-prep-provider-supplement-2021.pdf.

[5] FDA FDA Approves First Injectable Treatment for HIV Pre-Exposure Prevention. https://www.fda.gov/news-events/press-announcements/fda-approves-first-injectable-treatment-hiv-pre-exposure-prevention.

[6] CDC Ending the HIV Epidemic in the US (EHE). https://www.cdc.gov/ehe/index.html.

[7] CDC HIV in the U.S. by the Numbers—2021. NCHHSTP Newsroom. https://www.cdc.gov/nchhstp-newsroom/factsheets/hiv-in-us-by-the-numbers-2021.html.

[8] PrEP4All Toward PrEP Access for All: An Analysis of Policies, Approaches, and Strategies in the Southern United States. https://prep4all.org/publication/sac-report/.

[9] Sophus A.I., Mitchell J.W. (2019). A Review of Approaches Used to Increase Awareness of Pre-Exposure Prophylaxis (PrEP) in the United States. AIDS Behav..

[10] Berenbrok L.A., Tang S., Gabriel N., Guo J., Sharareh N., Patel N., Dickson S., Hernandez I. (2022). Access to Community Pharmacies: A Nationwide Geographic Information Systems Cross-Sectional Analysis. J. Am. Pharm. Assoc..

[11] Hogue M. CEO Blog. Pharmacists Expand Access to PrEP in 17 States. American Pharmacists Association. https://www.pharmacist.com/CEO-Blog/.

[12] Home|ClinicalTrials.gov. https://clinicaltrials.gov/.

[13] Ahmed K.K.M., Al Dhubaib B.E. (2011). Zotero: A Bibliographic Assistant to Researcher. J. Pharmacol. Pharmacother..

[14] Burns C.M., Endres K., Derrick C., Cooper A., Fabel P., Okeke N.L., Ahuja D., Corneli A., McKellar M.S. (2023). A Survey of South Carolina Pharmacists’ Readiness to Prescribe Human Immunodeficiency Virus Pre-exposure Prophylaxis. JAACP J. Am. Coll. Clin. Pharm..

[15] Crawford N.D., Josma D., Morris J., Hopkins R., Young H.N. (2020). Pharmacy-Based Pre-Exposure Prophylaxis Support among Pharmacists and Men Who Have Sex with Men. J. Am. Pharm. Assoc..

[16] Hopkins R., Josma D., Morris J., Klepser D.G., Young H.N., Crawford N.D. (2021). Support and Perceived Barriers to Implementing Pre-Exposure Prophylaxis Screening and Dispensing in Pharmacies: Examining Concordance between Pharmacy Technicians and Pharmacists. J. Am. Pharm. Assoc..

[17] Shaeer K.M., Sherman E.M., Shafiq S., Hardigan P. (2014). Exploratory Survey of Florida Pharmacists’ Experience, Knowledge, and Perception of HIV Pre-Exposure Prophylaxis. J. Am. Pharm. Assoc..

[18] Hoth A.B., Shafer C., Dillon D.B., Mayer R., Walton G., Ohl M.E. (2019). Iowa TelePrEP: A Public-Health-Partnered Telehealth Model for Human Immunodeficiency Virus Preexposure Prophylaxis Delivery in a Rural State. Sex. Transm. Dis..

[19] Chasco E.E., Shafer C., Dillon D.M., Owens S., Ohl M.E., Hoth A.B. (2021). Bringing Iowa TelePrEP to Scale: A Qualitative Evaluation. Am. J. Prev. Med..

[20] Khosropour C.M., Backus K.V., Means A.R., Beauchamps L., Johnson K., Golden M.R., Mena L. (2020). A Pharmacist-Led, Same-Day, HIV Pre-Exposure Prophylaxis Initiation Program to Increase PrEP Uptake and Decrease Time to PrEP Initiation. AIDS Patient Care STDs.

[21] Taliaferro T., Layson-Wolf C., Seung H., Banjo O., Tran D. (2021). Impact of Pharmacist-Led Program on Knowledge of College Students about Pre-Exposure Prophylaxis. J. Am. Pharm. Assoc..

[22] Nishimura M.J.K. (2024). FINISHING HIV: An Ending the HIV Epidemic (EHE) Model for Latinx Integrating One-Stop-Shop Pre-Exposure Prophylaxis (PrEP) Services, a Social Network Support Program and a National Pharmacy Chain; Clinical Trial Registration NCT06406049. NCT06406049.

[23] Crawford N. (2023). Advancing Pre-Exposure Prophylaxis (PrEP) Access in Pharmacies to Improve PrEP Uptake in Disadvantaged Areas; Clinical Trial Registration NCT04393935. NCT04393935.

[24] NASTAD Pharmacists’ Authority to Initiate PrEP and PEP and Engage in Collaborative Practice Agreements. https://nastad.org/resources/pharmacists-authority-engage-collaborative-practice-agreements-and-initiate-prep-pep-and.

[25] Cocohoba J., Tweedie L., Frank M., McElya B., Witt E. (2024). Legislation Expanding Pharmacist Scope of Practice to Furnish Human Immunodeficiency Virus Pre-Exposure Prophylaxis: A Content Analysis. JACCP J. Am. Coll. Clin. Pharm..

[26] Pellegrino A.N., Martin M.T., Tilton J.J., Touchette D.R. (2009). Medication Therapy Management Services: Definitions and Outcomes. Drugs.

[27] HIV Testing Sites & Care Services Locator. https://locator.hiv.gov/map.

[28] PrEP Locator: A National Database for US PrEP Providers. US PrEP Provider Directory. https://preplocator.org/.

[29] Gregory P.A., Austin Z. (2021). How Do Patients Develop Trust in Community Pharmacists?. Res. Soc. Adm. Pharm..

[30] Kolanczyk D.M., Merlo J.R., Bradley B., Flannery A.H., Gibson C.M., McBane S., Murphy J.A., Noble J.M., Noble M.B., Patton H.M. (2024). 2023 Update to the American College of Clinical Pharmacy Pharmacotherapy Didactic Curriculum Toolkit. J. Am. Coll. Clin. Pharm..

[31] American Pharmacists Association Pharmacy-Based HIV Prevention Services. https://www.pharmacist.com/Education/Certificate-Training-Programs/Pharmacy-Based-HIV-Prevention-Services.

[32] Saberi P., Su H., Mendiola J., Gruta C., Lutes E.R., Dong B., Bositis C., Chu C. (2024). HIV Pre-Exposure Prophylaxis Champion Preceptorship Training for Pharmacists and Nurses in the United States. JACCP J. Am. Coll. Clin. Pharm..

[33] Broekhuis J.M., Scarsi K.K., Sayles H.R., Klepser D.G., Havens J.P., Swindells S., Bares S.H. (2018). Midwest Pharmacists’ Familiarity, Experience, and Willingness to Provide Pre-Exposure Prophylaxis (PrEP) for HIV. PLoS ONE.

